# Factors in glucocorticoid regimens associated with treatment response and relapses of IgG4-related disease: a multicentre study

**DOI:** 10.1038/s41598-018-28405-x

**Published:** 2018-07-06

**Authors:** Mirei Shirakashi, Hajime Yoshifuji, Yuzo Kodama, Tsutomu Chiba, Motohisa Yamamoto, Hiroki Takahashi, Kazushige Uchida, Kazuichi Okazaki, Tetsuya Ito, Shigeyuki Kawa, Kazunori Yamada, Mitsuhiro Kawano, Shintaro Hirata, Yoshiya Tanaka, Masafumi Moriyama, Seiji Nakamura, Terumi Kamisawa, Shoko Matsui, Hiroto Tsuboi, Takayuki Sumida, Motoko Shibata, Hiroshi Goto, Yasuharu Sato, Tadashi Yoshino, Tsuneyo Mimori

**Affiliations:** 1 Department of Rheumatology and Clinical Immunology, Graduate School of Medicine, Kyoto University, Kyoto, Japan; 2 Department of Gastroenterology and Hepatology, Graduate School of Medicine, Kyoto University, Kyoto, Japan; 3 Kansai Electric Power Hospital, Osaka, Japan; 4 Department of Rheumatology and Clinical Immunology, Sapporo Medical University School of Medicine, Sapporo, Hokkaido, Japan; 5 Division of Gastroenterology and Hepatology, The Third Department of Internal Medicine, Kansai Medical University, Hirakata, Osaka, Japan; 6 Department of Gastroenterology, Shinshu University School of Medicine, Matsumoto, Japan; 7 Department of Internal Medicine, Matsumoto Dental University, Shiojiri, Japan; 8 Division of Rheumatology, Department of Internal Medicine, Kanazawa University Graduate School of Medical Science, Kanazawa, Ishikawa, Japan; 9 The First Department of Internal Medicine, School of Medicine, University of Occupational and Environmental Health, Japan, Fukuoka, Japan; 10 Department of Clinical Immunology and Rheumatology, Hiroshima University Hospital, Hiroshima, Japan; 11 Section of Oral and Maxillofacial Oncology, Division of Maxillofacial Diagnostic and Surgical Sciences, Faculty of Dental Science, Kyushu University, Fukuoka, Japan; 12 Department of Internal Medicine, Tokyo Metropolitan Komagome Hospital, Tokyo, Japan; 13 Health Administration Center, Sugitani Campus, University of Toyama, Toyama, Japan; 14 Department of Internal Medicine, Faculty of Medicine, University of Tsukuba, Tsukuba, Ibaraki, Japan; 15 Department of Ophthalmology, Tokyo Medical University, Tokyo, Japan; 16 Department of Pathology, Okayama University Graduate School of Medicine, Dentistry and Pharmaceutical Sciences, Okayama, Japan

## Abstract

Glucocorticoids (GC) are effective for treating IgG4-related disease (IgG4-RD); however, relapse is often observed. We conducted a retrospective multicentre study to investigate risk factors in GC regimens associated with relapses of IgG4-RD. Data on 166 patients with definitive IgG4-RD diagnosis were collected from 12 institutions. Comprehensive surveillance of clinical backgrounds and GC regimens as well as multivariate analysis of factors associated with treatment responses and relapses was performed. To determine the initial maximal GC dose, the patients were stratified into three groups depending on the initial prednisolone (PSL) dosage: <0.39, 0.4–0.69 and >0.7 mg/kg/day. The multivariate analysis extracted the disease duration and reduction speed of initial GC dose. Patients treated with initial GC <0.39 or >0.7 mg/kg/day of PSL showed higher relapse rates than those treated with 0.4–0.69 mg/kg/day. The relapse rates were significantly higher in patients with fast reduction of the initial dose (>0.4 mg/day) than in patients with slow reduction (<0.4 mg/day). To avoid relapse, 0.4–0.69 mg/kg/day of initial PSL with slow reduction speed (<0.4 mg/day) is needed in the early treatment of IgG4-RD.

## Introduction

IgG4-related disease (IgG4-RD) is a multiorgan sclerotic disease^1,2^ characterised by elevated serum IgG4 levels^3,4^ and infiltration of IgG4-positive plasma cells into target organs^2,5^. However, its etiology and pathophysiology have not been sufficiently elucidated. Hamano *et al*.^3^ reported high serum IgG4 levels in patients with autoimmune pancreatitis (AIP) in 2001, and several diseases such as Mikulicz disease^4^ have been also reported to be associated with IgG4. Consequently, a novel entity of IgG4-RD was established by integrating those diseases^1^. Patients with IgG4-RD respond to glucocorticoids (GC) effectively^6–10^, but relapse of the disease is observed frequently^8,10–13^. Because IgG4-RD mainly affects elderly people^3,10,13,14^, the long-term use of GC often harms these patients.

Although an initial dose of 0.6 mg/kg/day of prednisone or prednisolone (PSL) is often used to control IgG4-RD^8,9^, it is unclear if the initial dose and reduction speed of GC are suitable to control IgG4-RD. To refine the GC regimens, we performed a retrospective multicentre study on IgG4-RD. Because IgG4-RD is systemic, its study should not be biased by the specialties of the researchers. An outstanding point of this study is that a variety of specialists in gastroenterology, rheumatology, nephrology, dental and oral surgery, respiratory medicine, ophthalmology and pathology were involved.

## Results

### Patient profiles

**Figure 1:**
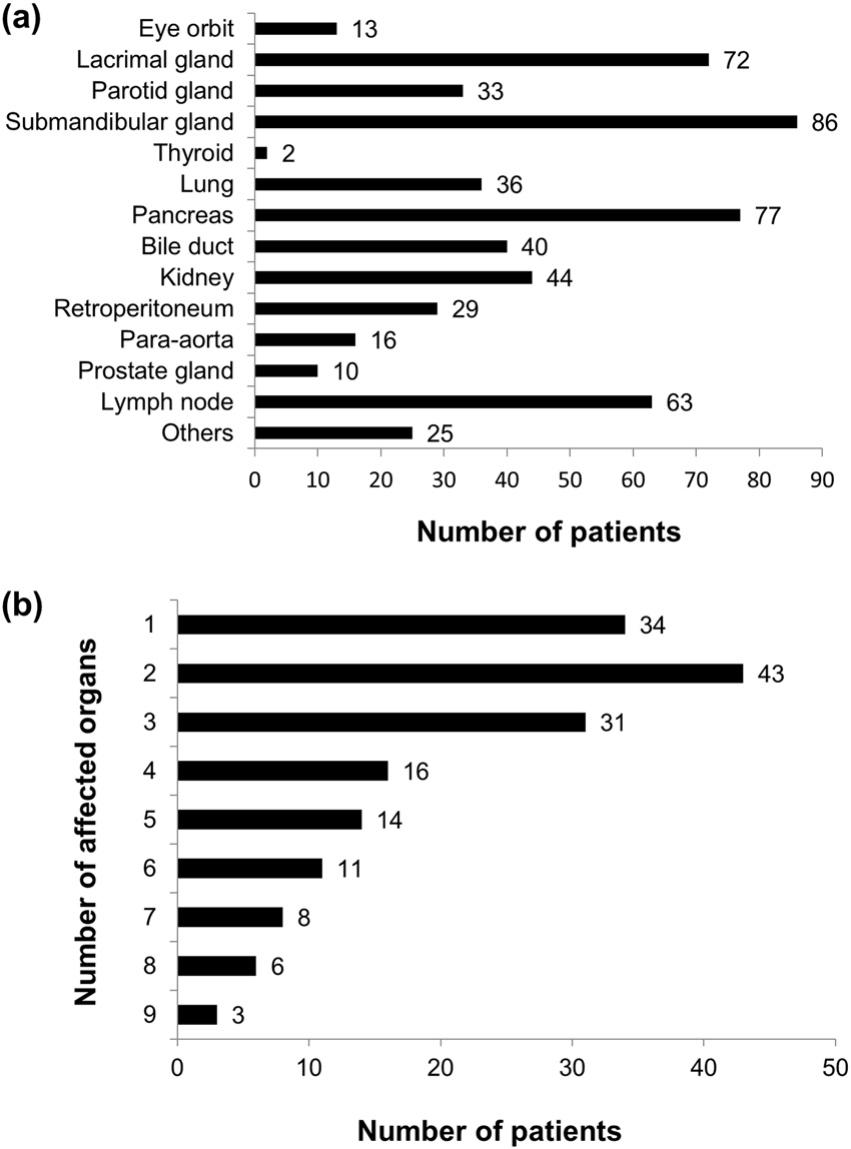
(**a**) Number of affected organs. The total number does not add up because some patients had multiorgan diseases. Others included the liver (5), pituitary gland (4), skin (4), nerve (3), mammary gland (2), vocal cord (1), colon (1), palate (1), paranasal sinus (1), vertebral body (1), epicardium (1) and urethra (1). (**b**) Number of affected organs by the number of patients.

Of the 166 patients (108 men, 65.1%; 58 women, 34.9%) who were evaluated, 140 fulfilled the comprehensive diagnostic criteria^15^ and 26 satisfied the Japan Pancreas Society’s diagnostic criteria for type 1 AIP^16^. The age at onset was 61.2 ± 11.8 years old (range: 16–85). The disease duration was 6.2 ± 3.8 years, and the observation period was 5.4 ± 3.4 years for the first comprehensive surveillance. Figure 1a shows the number of patients with every affected organ. The total number of organs did not add up to the number of patients because most patients had multiorgan disease, and 34 (20.5%) had a single-organ disease (Fig. 1b). The average number of affected organs was 3.3 ± 2.2 (Fig. 1b). The maximal serum IgG4 concentration was 847.4 ± 621.1 mg/dL. Four patients whose maximal serum IgG4 levels were under 135 mg/dL were diagnosed using the diagnostic criteria for AIP.

### Profiles of patients not treated with GC

Fourteen patients (8.4%) did not receive GC throughout the observation periods because they did not experience problems aside from cosmetic issues or because the lesions were removed surgically. Six patients had a single-organ disease. Two patients in whom an orbital tumour and pancreas were resected, respectively, were considered to be cured by surgery, and the disease did not recur.

### Response to GC and later relapse

**Figure 2:**
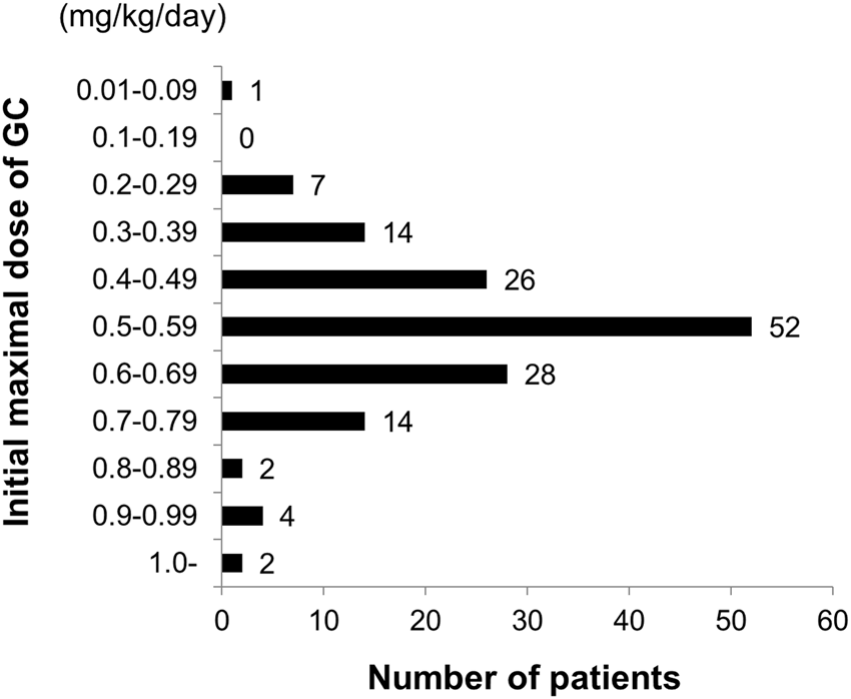
Distribution of the initial maximal doses of glucocorticoid (GC) per body weight (converted to prednisolone doses).

One hundred and fifty-two patients (91.6%) were treated with GC. The initial maximal GC dosage (PSL equivalent) per body weight was 0.55 ± 0.16 mg/kg/day in 150 patients whose data were available (Fig. 2). The response to GC could not be determined in 36 patients due to lack of clinical information. In the remaining 114 patients, 93 were responders (81.6%). According to the criteria of relapse, which are described in the Methods section, 46 (30.3%) of the 152 patients who were treated with GC experienced relapse. In the 46 relapsed patients, GC was increased in 42, steroid pulse therapy was performed in 2 and new immunosuppressants were added to GC in 2 patients. The peak GC dose after relapse was 21.6 ± 11.1 mg/day, excluding the 2 patients who received steroid pulse therapy.

### Immunosuppressants

In the first surveillance in 2014, 9 (5.9%) of the GC-treated patients received immunosuppressants. Six patients were treated with azathioprine (AZP) and the others received methotrexate, mizoribine or cyclosporine A. One, three and five patients were treated only with immunosuppressants without GC at the first surveillance, at 12 months and 24 months, respectively, and all of them received AZP (Supplementary Fig. S1).

### Factors associated with response and relapse

We compared the clinical backgrounds and GC regimens between the patients with and without treatment responses and relapses, respectively (Table 1). We selected the following factors: sex, disease duration, maximal serum IgG4 concentration, number of affected organs, reduction speed of initial GC dose, reduction rates in the first 3 and 6 months and initial maximal GC dose. The relapsed group had a significantly longer disease duration (P = 0.02) and significantly higher reduction speed of initial GC dose (P < 0.01). We analysed the relationship between the initial maximum GC dosage and relapse rate (Supplementary Fig. S2). We classified the patients into 3 groups based on the initial PSL dosage: 1) high dosage in general: >0.7 mg/kg/day, 2) intermediate dosage, including the recommended 0.5–0.6 mg/kg/day^15^: 0.4–0.69 mg/kg/day, and 3) low dosage: <0.39 mg/kg/day^17^. The rates of relapse among patients who were treated with GC <0.39, 0.4–0.69, and >0.7 mg/kg/day were 40.9%, 22.6%, and 54.2%, respectively (Table 1, right columns). Thus, the relapse rates of patients treated with an initial PSL dosage of <0.39 or >0.7 mg/kg/day were higher than those of patients treated with 0.4–0.69 mg/kg/day.

**Table 1:** Variables in patients with or without treatment responses and relapses.

	Treatment response		Relapse	
	Responder	Non-responder	Relapsed	Non-relapsed
	(n = 93)	(n = 21)	(n = 44)	(n = 106)
Male: female	67:26	15:6	33:11	68:38
Disease duration (years)	5.60 ± 3.46	4.81 ± 3.31	7.43 ± 4.53	5.81 ± 3.45
Maximal serum IgG4 concentration (mg/dL)	837.40 ± 557.94	831.05 ± 550.83	826.80 ± 556.77	862.81 ± 631.78
Number of affected organs	3.35 ± 2.19	3.10 ± 2.07	3.43 ± 1.85	3.30 ± 2.18
Reduction rate in the first 3 months	0.60 ± 0.18	0.64 ± 0.23	0.64 ± 0.18	0.59 ± 0.17
Reduction rate in the first 6 months	0.75 ± 0.15	0.72 ± 0.19	0.77 ± 0.16	0.74 ± 0.14
Reduction speed of initial dose of GC (mg/day)	0.34 ± 0.38	0.34 ± 0.33	0.46 ± 0.57	0.29 ± 0.19
Initial dose of GC (%)				
<0.39 mg/kg/day	64.50	35.50	40.91	59.09
0.4–0.69 mg/kg/day	85.90	14.10	22.64	77.36
>0.7 mg/kg/day	75.00	25.00	54.17	45.83

GC: glucocorticoid.

**Figure 3:**
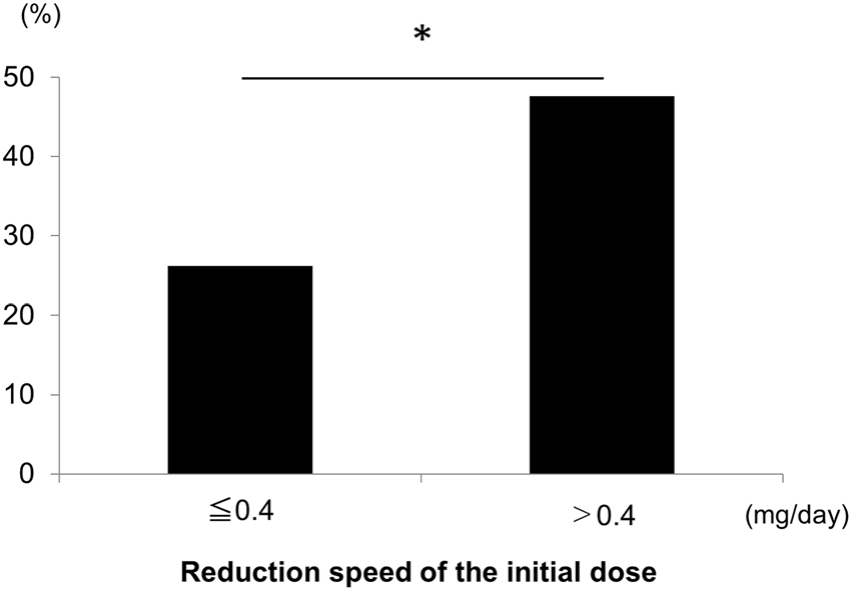
Relapse rates stratified by reduction speed of the initial dose. *P < 0.05 (Fisher’s exact test).

Next, we analysed the association of these variables with relapses by univariate and multivariate analyses (Table 2). In the univariate analysis, disease duration, reduction speed of initial GC dose, and initial dose of GC >0.7 mg/kg/day (ref: 0.4–0.69 mg/kg/day) were extracted. In the multivariate analysis, disease duration, reduction speed of initial GC dose, and initial dose of GC <0.39 and >0.7 mg/kg/day (ref: 0.4–0.69 mg/kg/day) were extracted. Figure 3 shows the relapse rates in patient groups stratified by the reduction speed of the initial dose. The relapse rate in patients with fast reduction of the initial dose (>0.4 mg/day) was 47.6%, which is significantly higher than that (26.2%) in patients with slow reduction of the initial dose (<0.4 mg/day) (p = 0.04).

**Table 2:** Univariate and multivariate analyses of variables associated with relapses.

Variable	Univariate analysis		Multivariate analysis	
	P value	OR (95%CI)	P value	OR (95%CI)
Male (%)	0.19	1.68 (0.78–3.82)	0.07	2.41 (0.94–6.90)
Disease duration (years)	0.02	1.11 (1.02–1.22)	0.01	1.14 (1.03–1.28)
Maximal serum IgG4 concentration (mg/dL)	0.74	1.00 (0.99–1.00)	0.23	1.00 (0.99–1.00)
Number of affected organs	0.73	1.03 (0.87–1.22)	0.34	1.11 (0.89–1.39)
Reduction rate in the first 3 months	0.11	5.68 (0.70–48.86)	NT	
Reduction rate in the first 6 months	0.22	4.75 (0.40–65.29)	NT	
Reduction speed of initial dose of GC (mg/day)	0.01	4.53 (1.41–19.4)	0.02	6.19 (1.22–46.93)
Initial dose of GC (%)				
<0.39 mg/kg/day	0.09	2.37 (0.68–6.18)	0.02	3.83 (1.25–11.75)
0.4–0.69 mg/kg/day	ref	ref	ref	ref
>0.7 mg/kg/day	<0.01	4.04 (1.61–10.35)	0.04	3.52 (1.09–11.51)

Patients treated with initial GC <0.39 and >0.7 mg/kg/day of PSL were compared with patients treated with 0.4–0.69 mg/kg/day of initial PSL. Only reduction speed of initial dose of GC, but not reduction rate in the first 3 or 6 months, was included in the multivariate analysis.

CI: confidence interval; GC: glucocorticoid; OR: odds ratio; NT: not tested; PSL: prednisolone; ref: reference.

The rates of responders among patients who were treated with GC <0.39, 0.4–0.69 and >0.7 mg/kg/day were 64.5%, 85.9% and 75.0%, respectively (Table 1, left columns). In the univariate and multivariate analyses, no variables associated with treatment response were extracted although low initial GC dose tended to be associated with non-responders (only the univariate analysis is shown in Table 3).

**Table 3:** Univariate analysis of variables associated with treatment responses.

Variable	P value	OR (95% CI)
Male (%)	0.95	1.03 (0.38–2.84)
Disease duration (years)	0.58	1.08 (0.93–1.27)
Maximal serum IgG4 concentration (mg/dL)	0.96	1.00 (0.90–1.00)
Number of affected organs	0.61	1.06 (0.85–1.35)
Reduction rate in the first 3 months	0.47	0.38 (0.02–5.11)
Reduction rate in the first 6 months	0.53	2.64 (0.13–52.78)
Reduction speed of initial dose of GC (mg/day)	0.96	1.04 (0.32–6.45)
Initial dose of GC (%)		
<0.39 mg/kg/day	0.05	0.30 (0.09–1.01)
0.4–0.69 mg/kg/day	ref	ref
>0.7 mg/kg/day	0.36	0.49 (0.13–2.45)

Patients treated with initial GC <0.39 and >0.7 mg/kg/day of PSL were compared with patients treated with 0.4–0.69 mg/kg/day of initial PSL.

CI: confidence interval; GC: glucocorticoid; OR: odds ratio; PSL: prednisolone; ref: reference.

Supplementary Fig. S3 shows how GC was used at the time of relapse. GC was stopped by the time of relapse in 17 (37.0%) of the 46 patients who experienced relapse. In the 46 patients, the dose of PSL was 2.9 ± 3.2 mg/day, whereas in 29 (63.0%) patients, it was <5 mg/day.

### Maintenance GC dose

In the first surveillance, 138 (90.8%) of the 152 GC-treated patients had a stable status and were either treated with maintenance dose or who had stopped GC (Supplementary Fig. S4). Thirty patients (21.7%) stopped GC, including 1 patient treated with AZP only. Forty-seven patients (34.1%) were treated with a maintenance GC dose <5 mg/day.

The average GC dose was 4.6–4.8 mg/day in the maintenance dose group at the 3 surveillance points (Supplementary Fig. S1). The 14 GC-untreated patients remained untreated at the 3 points.

### Remission maintenance curve

**Figure 4:**
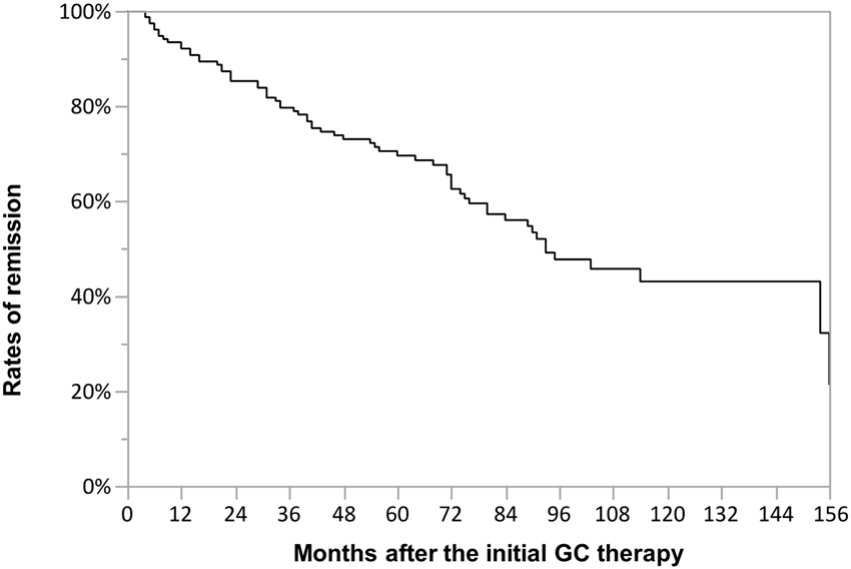
Remission maintenance curve. This indicates the remission rates after the initial glucocorticoid (GC) therapy in the 152 GC-treated patients, with a maximal observation period of 185 months.

The remission maintenance curve was plotted using the Kaplan-Meier method for the 152 GC-treated patients, with a maximal observation period of 185 months (Fig. 4). Relapse was observed in 8.0%, 14.8%, 27.1%, 37.5%, 52.4% and 57.1% of the patients at 12, 24, 48, 72, 96 and 120 months after the initial therapy, respectively.

### Outcomes and adverse events

In the first surveillance, 2 (1.2%) of 163 patients were found to have died, and the causes of death were infection and cancer. Two patients transferred to another hospital, and their outcomes were unknown. Two other patients had died at home of unknown causes at the time of the 24-month surveillance (Supplementary Fig. S1). Side effects of GC were seen in 78 (51.3%) of the 152 GC-treated patients; these included diabetes (27.0%), dyslipidaemia (17.8%), osteoporosis (9.9%), hypertension (6.6%), infection (3.9%), cataracts (3.9%), osteonecrosis (2.6%), glaucoma (2.0%), insomnia (1.3%), psychosis (1.3%) and compression fracture (0.7%).

## Discussion

We performed a retrospective, multicentre study on IgG4-RD to investigate risk factors in clinical backgrounds and GC regimens associated with relapses of IgG4-RD. A total of 84.3% of patients were definitively diagnosed with IgG4-RD based on the comprehensive diagnostic criteria of IgG4-RD^15^. Although IgG4-RD frequently occurs in the pancreas, biopsy of this organ has difficulties; therefore, we used the diagnostic criteria for AIP of the Japan Pancreas Society^16^, which included imaging findings and symptoms. We found that 79.5% of the patients had multiple lesions that affected various organs. Figure 1 shows that the selection of patients was well balanced among multiple specialty groups. The peak age at onset, sex ratio, high rate of GC responders, high relapse rate and remission maintenance curve in the present study were consistent with those of past reports^6–13^. The initial maximal GC dose per body weight (0.55 mg/kg/day) was slightly less than the 0.6 mg/kg/day that is recommended for treating AIP^8,9^.

We analysed the correlation between relapse and the initial maximal GC dose. Yamamoto *et al*.^12^ reported that a low GC dose at the initial treatment tended to be associated with relapse in IgG4-related dacryoadenitis and/or sialadenitis. In the present study, patients who were treated with <0.39 mg/kg/day of the initial PSL dose showed higher relapse rates than those treated with 0.4–0.69 mg/kg/day of initial PSL, suggesting that an initial dose of >0.4 mg/kg/day is appropriate. Paradoxically, the relapse rate of patients treated with >0.7 mg/kg/day of initial PSL was higher than that of patients treated with 0.4–0.69 mg/kg/day. This might be because a high dose of initial GC was selected in the treatment of atypically severe patients. The results imply that a high initial GC dose does not always lead to an improved relapse rate. Moreover, it leads to severer side effects of GC. Kamisawa *et al*.^8,9^ recommended 0.6 mg/kg/day for the treatment of AIP, whereas Umehara *et al*.^15^ recommended 0.5–0.6 mg/kg/day for IgG4-RD according to the guidelines for AIP. Saeki *et al*.^18^ divided patients with IgG4-related kidney disease (IgG4-RKD) based on the PSL dosage (>0.6 and <0.6 mg/kg/day) and showed that there were no differences between the two groups in terms of renal function at 1 month after the initiation of GC therapy and relapse rate within the first 3 years. The authors suggested that 0.5 mg/kg/day might be sufficient for the initial therapy of IgG4-RKD. In the present study, the relapse rates were constantly low at intervals of 0.40–0.49, 0.50–0.59 and 0.60–0.69 mg/kg/day (23%, 25% and 18%, respectively), and the rates of responders seemed favourable in these intervals. Taken together, the recommended initial GC dose for IgG4-RD is 0.4–0.6 mg/kg/day.

We found a significant correlation between relapse and speed of GC reduction at the initial therapy. The patients with fast reduction of the initial dose (>0.4 mg/day) were at risk of relapse. The reduction speed of 0.4 mg/day is equivalent to that of 5 mg PSL by 12.5 days after GC initiation. The duration of administration of the initial GC dose might suppress the activity of IgG4-RD and avoid relapse.

Regarding the maintenance dose, 90.8% of the GC-treated patients were treated with constant dose and about half of those patients took <5 mg/day in the first surveillance period. Similarly, in a Japanese database study by Yamamoto *et al*. (SMART)^13^, 92.1% of patients used a maintenance dose, and about half of them took <5 mg/day of GC. Kamisawa *et al*.^8^ reported that 82% of patients with AIP took a maintenance dose, and the majority took 5 mg/day of PSL. In the present study, 37.0% of the relapsed patients had already stopped GC by the time of recurrence. In the relapsed patients, the GC dose at the time of relapse was 2.9 ± 3.2 mg/day, similar to the 3.2 ± 3.7 mg/day in the SMART registry^13^. Ghazale *et al*.^19^ reported that 53% of GC-discontinued patients experienced relapse of IgG4-associated cholangitis. Masamune *et al*.^20^ recently performed a multicentre randomised controlled trial using 49 patients with AIP and reported that patients who discontinued GC experienced relapse significantly more often (58%) than patients with maintained GC (23%, most took PSL 5–7.5 mg/day) in a 36-month observation period. Altogether, careful observation is needed when the GC dose is reduced below 5 mg/day.

In the present study, 7.9% of patients were treated with immunosuppressants, which is consistent with the rate of 9.0% in the SMART study^13^. In Japan, a maintenance GC dose tends to be used for relatively long periods, and immunosuppressants are used for a limited number of cases^21^ compared with the high rates (34.5–48%) of immunosuppressant use in European studies^22,23^.

The low mortality (1.2%) in the average observation period of 5.4 years and high frequency (51.3%) of side effects related to GC in the present study were consistent with rates of 3.6–4.0%^22,23^ and 67%^22^, respectively, in the European reports. The causes of death were not directly attributed to IgG4-RD in either the European studies or ours, although they were rather attributed to the side effects of GC.

This study has several limitation because of its retrospective nature. The responders and relapses were determined using information from the patients’ medical records. The proposed definition of responders and relapses of IgG4-RD that was used in this study should be validated in a prospectively designed study. Recently, Masaki *et al*.^24^ conducted a prospective study of IgG4-RD (44 patients had a definite diagnosis), in which they used a unified GC dose. They started the trial with an initial dose of 0.6 mg/kg/day of PSL and reported a good response rate (93.2%). They reduced the dose of PSL by 10% biweekly and used a maintenance dose of 10 mg of PSL for at least 3 months. The relapse rate in their study was low as 14.6%, suggesting that standardised prospective regimens will bring favourable outcomes.

In conclusion, careful and slow (<0.4 mg/day) tapering of GC is needed in the early treatment of IgG4-RD and when the GC dose is reduced below 5 mg/day.

## Methods

### Patients

One hundred and sixty-six patients who were definitively diagnosed with IgG4-RD through the comprehensive diagnostic criteria of IgG4-RD^15^ or the diagnostic criteria for type 1 AIP of the Japan Pancreas Society^16^ were collected from 12 institutions (13 departments) belonging to the Research Committee for the Study of Pathophysiology and Development of New Treatments in IgG4-RD of the Japan Agency for Medical Research and Development. All analyses were performed in accordance with approved guidelines and regulations. Because this study is a non-invasive retrospective observational study, informed consent of patients was obtained in the opt-out way by disclosing information of the study on the internet. In advance, approvals of the study protocol including patient recruitment were acquired by ethical committees of all the institution. Clinical information was collected from the patients’ medical records. The cases were selected serially in each institution. The first comprehensive surveillance of outcomes, GC dose and use of immunosuppressants was performed in 2014. These same factors were surveyed again 12 and 24 months later.

### Definition of responders and relapse

A responder to therapy was defined as a patient who fulfilled one or more of the following items 4 weeks after initiation of the therapy: (1) improvement of organ function disorders such as decreased tear or salivary secretion, biliary obstruction and hydronephrosis; (2) the reduction of the target mass/organ’s diameter was >50% on physical examination, computed tomography (CT), magnetic resonance imaging (MRI) or ^18^F-fluorodeoxyglucose positron emission tomography CT (^18^F-FDG-PET-CT); or (3) the reduction of the target mass/organ’s diameter was >10% on physical examination, CT, MRI or ^18^F-FDG-PET-CT, and the reduction of serum IgG4 was >30%. If the patient has multiple organ involvements, he or she is regarded as a responder when the most prominent lesion fulfills the above criteria. Relapse of the disease was retrospectively determined when (1) any emerging or worsening of existing organ function disorders, organ swelling or mass-forming lesions were detected on physical examination, CT, MRI, or ^18^F-FDG-PET-CT and (2) the GC dose was increased or (3) an immunosuppressant was newly added to the GC regimen to control the emerging or worsening symptoms of IgG4-RD.

To evaluate the reduction speed of GC, we surveyed (a) the initial GC dose (mg), (b) the GC dose (mg) 3 months after initiation, (c) the GC dose (mg) 6 months after initiation, (d) the change at the first reduction from the initial GC dose (mg) and (e) the duration of the initial GC dose that was kept unchanged (days). Three parameters were defined as follows: (1) the rate of the reduction in the first 3 months: (*a* − *b*)/*a*, (2) the rate of the reduction in the first 6 months: (*a* − *c*)/*a* and (3) the speed of the reduction of the initial dose: *d*/*e* (mg/day).

The GC doses were converted to equivalent PSL doses.

### Statistical analysis

Student t-test was used for the comparison of unpaired groups. Variables were described as mean (±S.D.). In univariate and multivariate logistic regression analyses, results were expressed as odds ratios (OR) with the 95% confidence interval (CI). The remission maintenance curve was plotted using the Kaplan-Meier method. P < 0.05 was considered to be statistically significant. Statistical analyses were performed with JMP® software (SAS Institute Inc., Cary, NC, USA).

### Data availability

All data generated or analysed during this study are included in this published article and its Supplementary Information files.

## Electronic supplementary material

Supplementary Dataset 1
