# Supplementary material for: Factors in glucocorticoid regimens associated with treatment response and relapses of IgG4-related disease: a multicentre study
Source: Sci Rep. 2018 Jul 6;8:10262. doi: 10.1038/s41598-018-28405-x (PMC6035219; doi:10.1038/s41598-018-28405-x)
Supplement: Supplementary file 1 — Supplementary Dataset 1 [file 41598_2018_28405_MOESM1_ESM.pdf]

Supplementary Figures

Title

Factors in glucocorticoid regimens associated with treatment response and relapses of IgG4-related disease: a multicentre study

Authors

Mirei Shirakashi, Hajime Yoshifuji, Yuzo Kodama, Tsutomu Chiba, Motohisa Yamamoto, Hiroki Takahashi, Kazushige Uchida, Kazuichi Okazaki, Tetsuya Ito, Shigeyuki Kawa, Kazunori Yamada, Mitsuhiro Kawano, Shintaro Hirata, Yoshiya Tanaka, Masafumi Moriyama, Seiji Nakamura, Terumi Kamisawa, Shoko Matsui, Hiroto Tsuboi, Takayuki Sumida, Motoko Shibata, Hiroshi Goto, Yasuharu Sato, Tadashi Yoshino and Tsuneyo Mimori

Supplementary Figure S1.

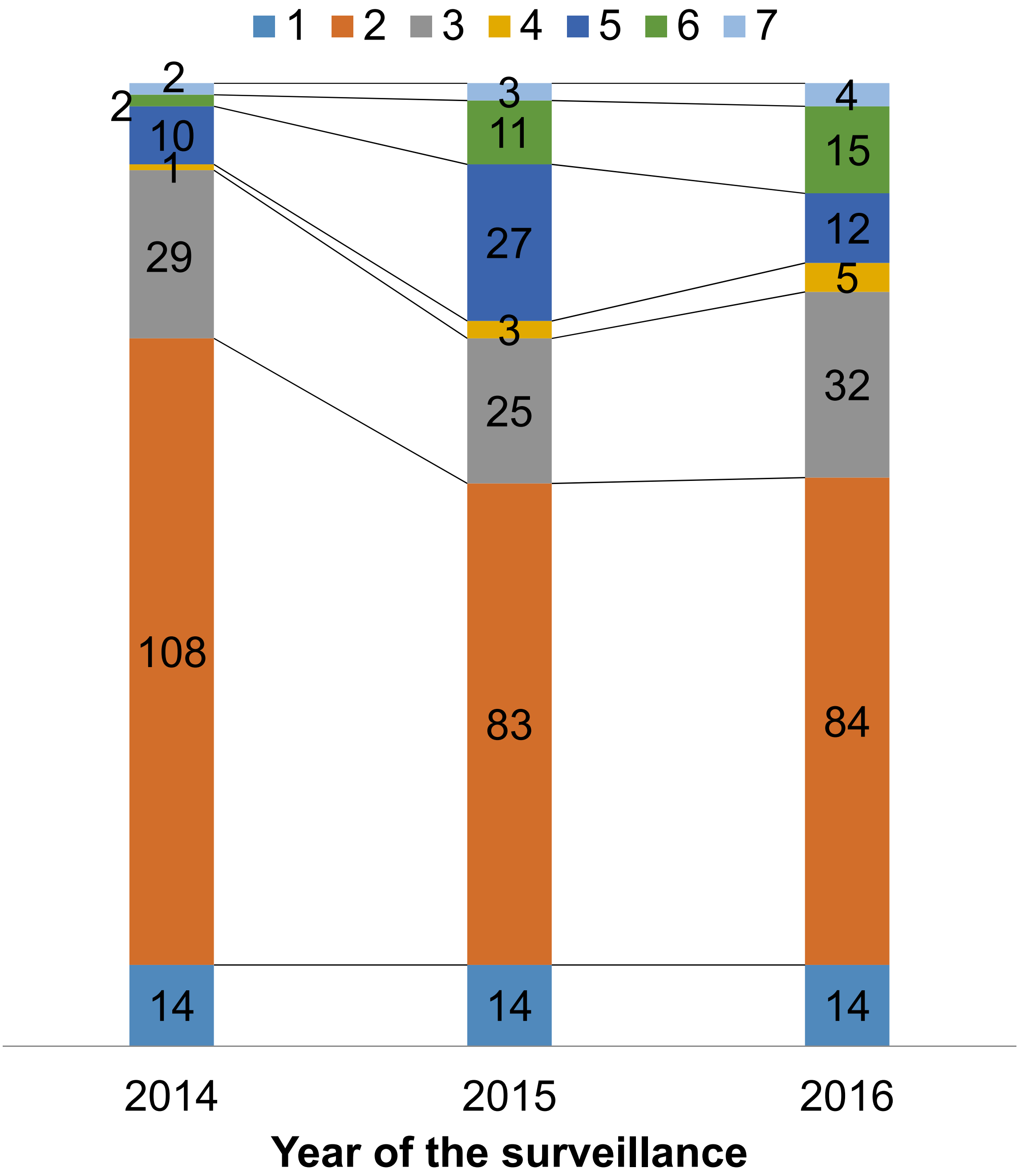

Supplementary Figure S1.
The state of treatments and outcomes of 166 IgG4-RD patients at the surveillances in 2014, 2015 and 2016. (1) Untreated, (2) remission with prednisolone (PSL), (3) drug-free remission, (4) remission with only immunosuppressants other than PSL, (5) recurrence, (6) hospital transfer or dropout and (7) deceased.

Supplementary Figure S2.

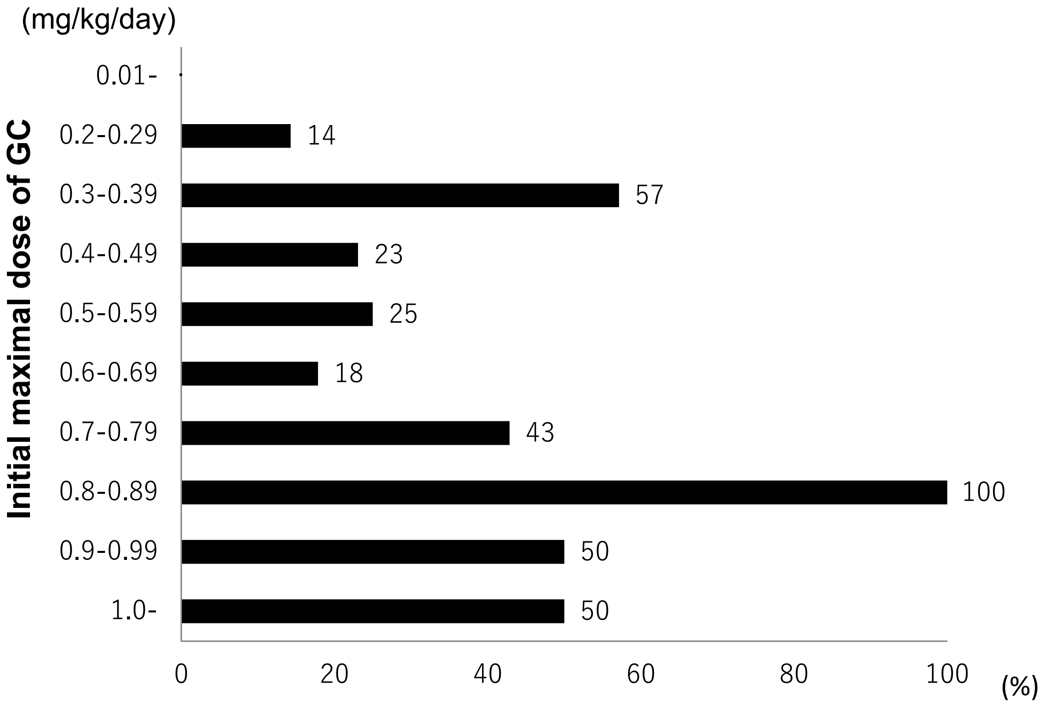

Supplementary Figure S2.
Relapse rates stratified by initial maximal dose of glucocorticoid (GC).

Supplementary Figure S3.

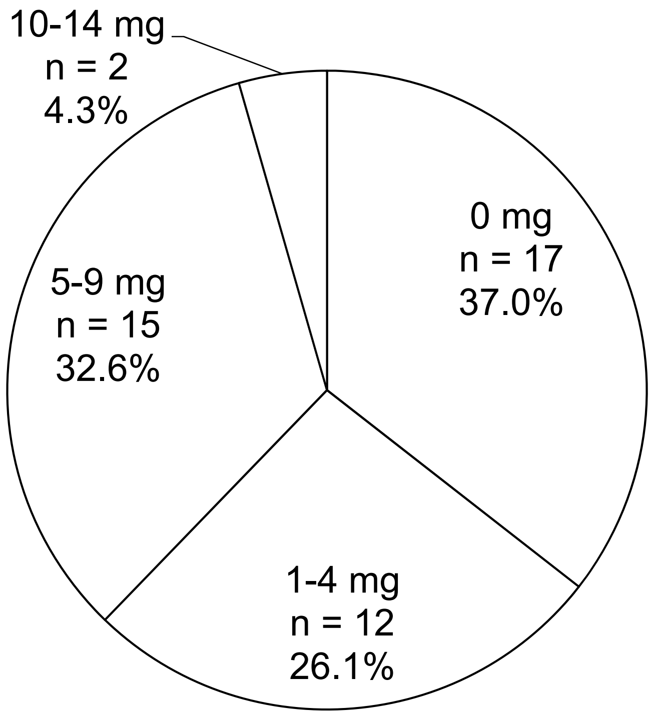

Supplementary Figure S3.
Dose of glucocorticoid (GC) at the time of relapse in the 46 relapsed patients. GC was stopped by the time of relapse in 17 of the relapsed patients.

Supplementary Figure S4.

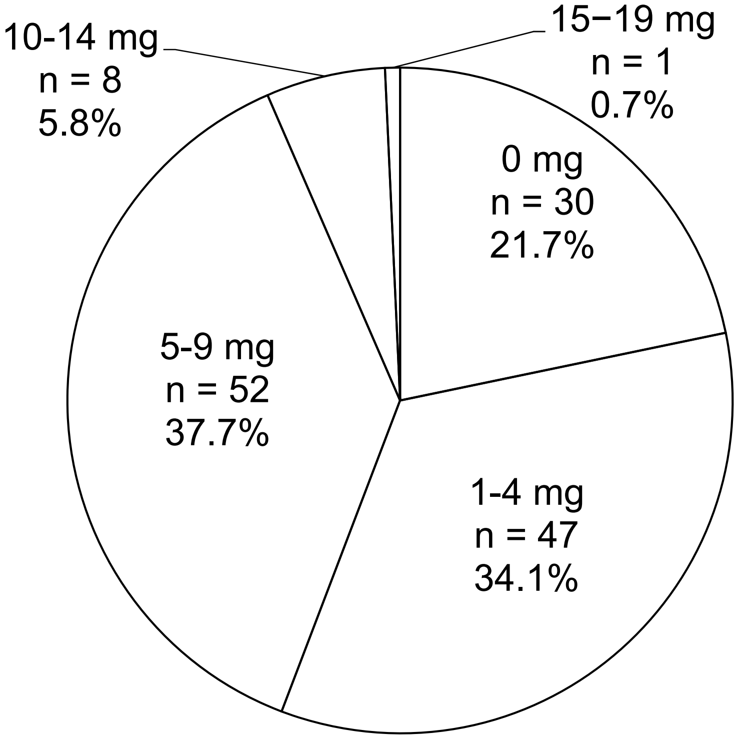

Supplementary Figure S4.
Dose of glucocorticoid (GC) in 138 patients were treated with constant dose and about half of those patients took <5 mg/day in the first surveillance period.
